# Effect of lacquer decoration on VOCs and odor release from *P. neurantha* (Hemsl.) Gamble

**DOI:** 10.1038/s41598-020-66724-0

**Published:** 2020-06-12

**Authors:** Qifan Wang, Bin Zeng, Jun Shen, Huiyu Wang

**Affiliations:** College of Material Science and Engineering, Northeast Forestry University, Harbin, China

**Keywords:** Environmental sciences, Materials science

## Abstract

The problem of odor caused by solid wood and its lacquer finish is increasingly serious. In this study, gas chromatography–mass spectrometry/olfactometry is used to analyze the volatile organic compounds and odor-active substances released from *Phoebe neurantha* (Hemsl.) Gamble lacquered with three types of lacquers, which is helpful in solving furniture’s odor problem. The results show that olefin emission of the three types of lacquer coating for *P. neurantha* (Hemsl.) Gamble decreases by more than 90% but that total volatile organic compound release increases. Among these, polyurethane (PU) lacquer could lead to a sharp increase of ester and aromatic hydrocarbons. Waterborne lacquer also releases numerous esters and alcohol compounds. Ultraviolet (UV)-curable lacquer has the greatest inhibitory effect on alcohols, aldehydes, and ketones and does not release esters or other compounds, but the release of toluene increases sharply. Benzaldehyde, toluene, and 1,3-dimethylbenzene are identified as key odor characteristic compounds of *P. neurantha* (Hemsl.) Gamble. Aromatic and fruity are the main odor characteristics of *P. neurantha* and three types of lacquer decoration studied. The overall odor intensity increases with lacquer treatment, especially PU lacquer. Among them, UV lacquer has the lowest overall odor intensity.

## Introduction

With the improvement of human standards of living, people are paying increasing attention to the indoor air environment. The main source of indoor air pollution is volatile organic compounds (VOCs)^1,2^, which are regarded as hidden killers in decoration by the medical community, and the odor produced by some VOCs has become a common sensitivity for some people^3^. VOCs can lead to serious harm to human health^4^ When the concentration of VOCs in an indoor environment reaches a certain level, people will feel sick, experiencing headache, nausea, vomiting, fatigue, and other symptoms. When the effects are serious, people may have convulsions; enter a coma; experience effects on their mind, blood circulation, liver, kidneys, etc.; and even suffer from leukemia^5^. Wallace^6^ found that VOCs in benzene, dichloroethylene, dichlorobenzene, dichloromethane, carbon tetrachloride, and other organic compounds have certain genotoxicity and carcinogenicity, which are among the main causes of the sick building syndrome^7^. In addition, for a period after a house has been decorated, residents often perceive an obvious chemical smell in a room, even when testing shows the VOCs do not exceed the standard acceptable level. This is because some compounds can produce a peculiar smell even when the concentration is lower than the limit value of the existing standard. Therefore, humans living in odor pollution environment for a long period may experience multiple effects. The odor environment can affect human health, such as stimulation of eyes, nose, respiratory tract, and skin; central nervous system abnormality; and functional obstacles of heart, liver, kidneys, spleen, and hematopoiesis, and also will harm the human spirit, leading to a series of problems such as emotional restlessness, difficulty in concentrating, energy at work, and inability to sleep normally^8^.

Among the many types of furniture, solid wood furniture is popular because of its unique texture and comfortable nature. However, furniture materials need to be covered with various coatings to decorate and protect the wood. Lacquer can give wood color and improve gloss and smoothness, as well as enhance the three-dimensional nature and touch sense of the wood’s texture. At the same time, properties of coated wood such as moisture, water, and oil resistance will be improved to varying degrees.

At present, the odor of wood has been well investigated, but research is still not extensive. Yang *et al*.^9^ proposed two feasible odor detection schemes for solid wood furniture, such as sampling and tracking the production process of furniture odors based on consumer complaints. The composition of odors emitted from a solid wood bedside cabinet were also studied^10^. It was found that the benzene series and a few low molecular lipids, such as *ortho*-*para*-xylene (*o*-*p*-xylene), *n*-butyl acetate, and *sec*-butyl acetate, were the main components of odors. Liu *et al*.^11^ found that ethanol–toluene solution extraction could reduce the intensity of some odors of poplar (*Populus cathayana* Rehd.) and rubber (*Hevea brasiliensis* (Willd. ex A. Juss.) Muell. Arg.) but that residual benzene was produced during extraction, which led to an increase in benzene odor. Wang *et al*.^12^ studied the odor compounds released from poplar (*Populus ussuriensis* Kom.), pine (*Pinus sylvestris* L. var. *mongholica* Litv.) and linden (*Tilia amurensis* Rupr.); the key odorants were identified by gas chromatography–mass spectrometry/olfactometry (GC-MS/O). Schreiner *et al*.^13^ studied odor-active compounds released from *P. sylvestris* L. var. *mongholica* Litv. in Germany by gas chromatography–olfactometry (GC-O) and aromatic extract dilution analysis, identifying 44 types of odorous compounds. Ghadiriasli *et al*.^14^ found that the odor of oak wood mainly came from fatty acid degradation products, terpenoids, and lignin degradation based on GC-O and odor extraction dilution analysis technology. It was found that most odor components of oak were composed of a series of terpenoids, mainly monoterpenes and sesquiterpenes, aldehydes, acids and lactones, and some polyphenols containing phenolic core components.

In practical application, wood used indoors mostly features lacquer decoration, but there are few reports on this aspect. Wang *et al*.^15,16^ investigated the effects of environmental factors on particleboard with different lacquers and found the temperature, relative humidity, and ratio of the air exchange rate to the loading factor have different influences on lacquered boards during the release process, but the differences among various particleboards with different lacquers was not showed. There are also few reports about the odor emission from solid wood with lacquer.

In this study, the odor-active substances released from *Phoebe neurantha* (Hemsl.) Gamble, which is widely used as a furniture material for its characteristics of strong corrosion resistance and sturdiness^17^ were analyzed by GC-O technology. In addition, the odor and VOCs of *P. neurantha* with different commonly used lacquers, namely, polyurethane (PU) coatings, waterborne coatings, and ultraviolet (UV)-curable coatings, were investigated and compared. *P. neurantha*can release VOCs and odors into the surrounding environment during production, display, and use, which can affect indoor air quality. *P. neurantha* can also release its unique aroma, which prevents intrusion by insects and disperses mosquitoes. However, the odor released by the boards changes after a lacquer coating is applied, because the odor released from the wood and the surface coating have different interactions. Therefore, to better understand odor release from coated *P. neurantha*, it is necessary to study *P. neurantha* coated with different lacquers.

## Results and Discussion

### Analysis of TVOC and VOC Components from *P. neurantha* with different lacquers

**Figure 1:**
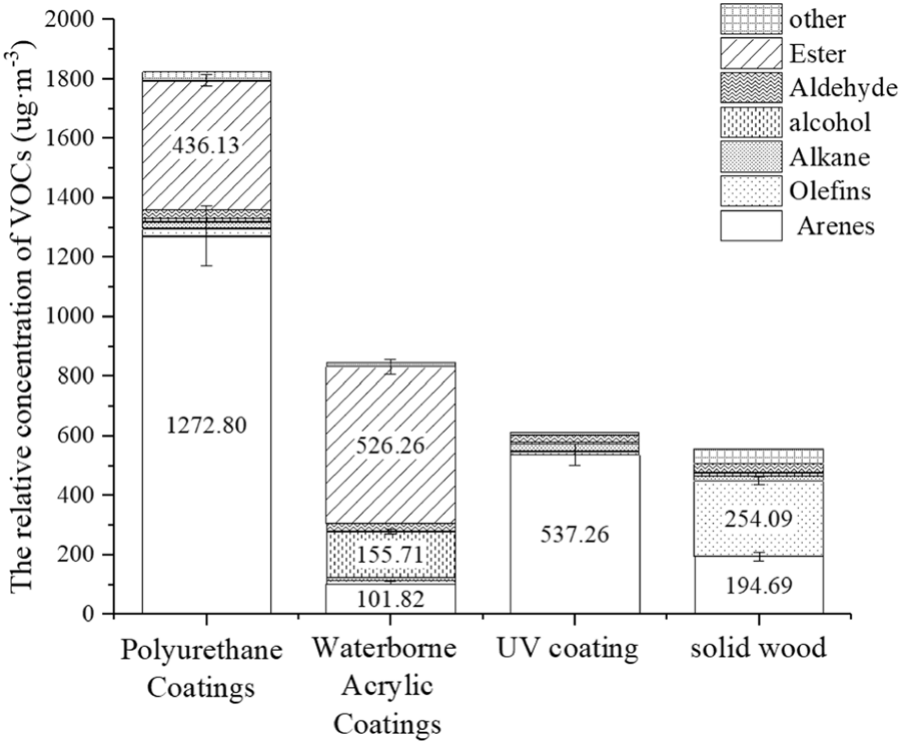
Relative concentration of VOCs from *P. neurantha (Hemsl.) Gamble* with three lacquer coatings.

In this study, the release of VOCs from *P. neurantha* and three types of lacquered boards was analyzed. Total volatile organic compound (TVOC) emission of *P. neurantha* increased after it was decorated with one of three types of lacquer (Fig. 1). The major constituents of *P. neurantha* were aromatics and olefins. A few alkanes, alcohols, aldehydes, and other compounds were also found, but esters compounds were not detected. After decoration, the release of VOC components from solid wood changed drastically. Aromatic hydrocarbons and esters were the main constituents of PU-lacquered *P. neurantha*, compared with the primary compounds of esters from waterborne-lacquered *P. neurantha*, and main components of aromatic hydrocarbons (accounting for 87.7% of the TVOC) from UV-lacquered *P. neurantha*. It was remarkable that the VOC concentration increased greatly after PU lacquer decoration and the TVOC increased by about 227.9%. Wang *et al*.^18^ came to a similar conclusion indirectly. They studied the VOCs in the air of a PU synthetic leather factory using an adsorption tube and a secondary thermal desorption GC mass selective detector and found the PU would release a great quantity VOCs.

After being decorated with lacquer, some VOCs released from solid wood are inhibited; however, the lacquer material releases other compounds at the same time. The inhibitory effects on VOC components released from *P. neurantha* varied among the lacquer decorations. Table 1 shows the increase rate of TVOC and VOC constituent concentration of *P. neurantha* with three types of lacquer coating. Research showed that the olefins from *P. neurantha* were inhibited by these three types of lacquers (the inhibition rate was more than 90%).

**Table 1:** The release increase rate of tvoc and components.

The Release increase rate of tvoc and components under different paints							
Types of Paints	TVOC/%	Aromatic /%	Olefins/%	Alkanes/%	alcohols/%	Aldehydes/%	others/%
Polyurethane Coatings	227.9	553.74	−90.59	41.13	1.45	−7.79	−37.52
Waterborne Acrylic Coatings	51.78	−47.7	−95.63	−42.75	1251.71	−9.88	−72.26
UV coating	9.95	175.95	−95.53	37.1	−39.28	−26.49	−76.52

PU lacquer could inhibit aldehydes, ketones, and other compounds, but the release of aromatic hydrocarbons increased sharply, with an increase rate of 553.74%, and 436.13 µg·m^−3^ of esters were found from the PU lacquer. Among them, 1,3-dimethylbenzene, *o*-xylene, and butyl acetate increased most significantly, with concentrations reaching 633.85, 240.12, and 273.71 µg·m^−3^, respectively. These three compounds had different effects on humans. Correlation research indicated that 1,3-dimethylbenzene would irritate human eyes and the upper respiratory tract; it also had anesthetic effect on the central nervous system at a high concentration. The lowest toxic concentration of 1,3-dimethylbenzene inhaled in rats was 3000 mg·m^−3^ (24 h)^19^, which was much higher than the release amount found in this experiment. Residue and accumulation of this compound were not serious, and photolysis might happen when it is transferred to the atmosphere^20^, giving it slight toxicity; however, long-term exposure could lead to neurasthenia syndrome, dry and chapped skin, dermatitis, etc. As a low-toxicity compound, *o*-xylene could stimulate the skin and mucosa and anesthetize the central nervous system. The lowest toxic concentration of inhalation in rats was 1500 mg·m^−3^. Although the release detected in this experiment was far less than the toxicity value, long-term exposure still would affect the function of liver and kidneys, and severely affected patients might even have hallucinations and unconsciousness^21^. Butyl acetate has slight toxicity^22^, but it could stimulate the eyes and upper respiratory tract. Long-term inhalation of butyl acetate at a high concentration might lead to tears, a sore throat, coughing, chest tightness, shortness of breath, and other symptoms. Serious cases could experience conjunctivitis and keratitis, and skin contact could cause dry skin^23^.

Among these three lacquers, the lowest amount of VOCs were released from *P. neurantha* varnished with UV (they only increased about 10%). It had the greatest inhibitory effect on olefins, alcohols, aldehydes, and ketones and did not release esters or other compounds. However, the release of toluene from *P. neurantha* varnished with UV increased sharply, reaching 403.36 µg·m^−3^, nearly 33.5 times that of unpainted *P. neurantha*. According to the relevant data, toluene has high toxicity^24^. At a certain concentration, it can stimulate human skin and mucosa and has an anesthetic effect on the central nervous system. Therefore, toluene should be the key limiting substance in VOCs released from indoor wood furniture. In China, the concentration limit of toluene released from wood-based panels and their products for interior decoration was less than 100 µg·m^−3^. The U.S. Business and Institutional Furniture Manufacturers Association stipulated that the toluene released from seats should not higher than 250 µg·m^−3^. Japanese industrial standards also set the limit that the toluene in indoor air should less than 260 µg·m^−3^. In this study, the mass concentration of toluene exceeded the standard; therefore, although UV lacquer has a good inhibitory effect on VOC release, the concentration of toluene released is high, which would influence the indoor environment. Therefore, to control the emission of VOCs more comprehensively, it is suggested the emission of toluene in UV lacquer should be controlled.

The TVOC concentration of waterborne-lacquered boards was 233.16 µg·m^−3^ higher than that of UV-lacquered boards. It could inhibit the release of aromatic hydrocarbons, olefins, alkanes, aldehydes, and ketones, but the release of alcohols increased by 12.5 times, and it released 526.25 µg·m^−3^ of esters. Among them, 2,2′-oxybis-1-propanol, 3,3′-oxybis-1-propanol, and 2-methyl-propanoic acid-1-(1,1-dimethylethyl)-2-methyl-1,3-propanediyl ester increased most significantly, which had little impact on humans. Above all, compared with PU and UV, waterborne-lacquered boards were less harmful to humans.

### Characterization of odor-active compounds of *P. neurantha* with different lacquers

Based on GC-MS library search, GC-O olfaction, and retention index analysis, 19 types of odor-active compounds were identified from four kinds of samples and were classified as alcohols (2 substances), aromatic hydrocarbons (6 substances), aldehydes (5 substances), or esters (6 substances). The specific odor characteristic compounds are shown in Table 2.

**Table 2:** Characteristic odor compounds and concentrations released from four samples.

Mass concentration of odorant compounds released from three paint finishes and solid wood							
Serial	Molecular Formula	Compound Name	Odor Characteristics	Mass Concentration /ug·m^−3^			
				Polyurethane lacquer	Waterborne Acrylic lacquer	UV lacquer	Solid wood
1	C_6_H_6_	benzene	Aromatic	—	18.2939	23.1977	12.8192
2	C_7_H_8_	toluene	Aromatic, sweet	16.3181	12.3449	403.3592	11.6758
3	C_8_H_10_	ethylbenzene	Aromatic, sweet	176.5671	16.5416	26.5617	25.3815
4	C_8_H_10_	1,3-dimethyl-benzene	Aromatic	633.8477	49.6477	74.6074	93.3388
5	C_8_H_10_	o-xylene	Aromatic	240.1235	—	—	—
6	C_9_H_12_	1,2,3-trimethyl-benzene	Aromatic	73.0694	—	—	—
7	C_6_H_12_O	2-ethyl-cyclobutanol	Cheese flavor	5.7164	—	—	—
8	C_6_H_12_O_2_	acetic acid, butyl ester	Fruity	273.7115	—	—	—
9	C_6_H_12_O	hexanal	Green grass scent	6.6422	—	—	9.2318
10	C_7_H_6_O	benzaldehyde	Almond	—	5.1650	—	4.9284
11	C_8_H_16_O	octanal	Fruity sweet and sour	5.2479	5.6745	5.4471	4.5176
12	C_8_H_18_O	2-ethyl-1-hexanol,	Sweet flower	5.9694	8.7467	6.9941	11.5193
13	C_9_H_18_O	nonanal	Fruity	9.0897	8.6742	5.9350	6.2410
14	C_10_H_20_O	decanal	Citrus smell	7.3752	8.1977	5.4382	5.8315
15	C_7_H_14_O_2_	3-methyl-2-butanol,acetate	Fruity, sweet and sour	6.8742	—	—	—
16	C_7_H_12_O_4_	pentanedioic acid,dimethyl ester	Light fragrance	17.5278	—	—	—
17	C_7_H_14_O_2_	2-pentanol, acetate	Fruity aroma	17.7815	—	—	—
18	C_6_H_12_O_2_	acetic acid, 2-methylpropyl ester	Aromatic	8.3519	5.5885	—	—
19	C_16_H_22_O_4_	dibutyl phthalate	Light aromatic	7.4099	4.7528	—	—

Aromatic hydrocarbons and aldehydes were the main characteristic odor compounds of *P. neurantha*, and aromatic was the main odor characteristic according to the results of olfactory identification, which played a major role in the overall odor formation of *P. neurantha*. The odor characteristics of *P. neurantha* were identified as follows: benzene was reported as aromatic, the same as the finding of aromatic odor by the U.S. National Institute for Occupational Safety and Health (NIOSH)^25^. Sax^26^ also found it to have a gasoline-like and rather pleasant aromatic odor, and the burnt characteristic was reported by Wang *et al*.^27^. Toluene was found to have both an aromatic and a sweet characteristic; the characteristic of sweet and pungent was reported by NIOSH^25^. Our testing found that ethylbenzene has a sweet and pungent odor, similar to the sweet and gasoline-like odor reported by the U.S. National Oceanic and Atmospheric Administration^28^; it was also found to be aromatic by Larranaga *et al*.^29^. The hexanal detected in this experiment had a green grass characteristic, the same as was detected by Furia^30^, whereas its odor was also described as fruity by Burdock^31^ and sharp and aldehyde by Lewis^32^. The benzaldehyde in this experiment was reported to have an almond character, which was also found in the research of O’Neil^33^ and Larranaga *et al*.^29^. In this experiment, octanal was reported to have a fruity sweet-and-sour smell, similar to the fruity odor reported by Larranaga *et al*.^29^. Other researchers found it to present a fatty, citrus, and honey odor^31^ and a pungent odor^34^. The nonanal was found to be fruity in this research, which was also found to be an orange–rose odor by Lewis^32^ and to have a floral, waxy, and green character by Nishimura^35^. Decanal was reported as having a citrus smell in this study, similar with the orange peel found by Kohlpaintner *et al*.^36^, whereas Lewis^32^ reported a slight floral–fatty odor. The odor characteristic of 2-ethyl-1-hexanol (sweet flower) and 1,3-dimethylbenzene (aromatic) were reported for the first time.

After varnishing with PU lacquer, the odorous compounds increased to 17 substances, including nine types of new odor compounds. The odor compounds released from PU lacquer-coated *P. neurantha* had the greatest number of substances among these three types of lacquers, and the concentration of odor compounds was significantly higher than for other boards. Among them, 2-ethyl cyclobutanol (cheese odor), butyl acetate acetic acid (fruit odor), 3-methyl-2-butanol acetate (sweet-and-sour fruit odor), 2-pentanol acetate (fruit odor), and 1,2,3-methylbenzene (aromatic) had an important contribution to the overall odor formation of board. Therefore, compared with *P. neurantha*, the overall odor characteristics of PU lacquer-coated board were significantly different. The odor intensity of PU lacquer-coated *P. neurantha* was stronger than that of uncoated board. The odor compounds of waterborne lacquer-coated *P. neurantha* were mostly similar to unpainted *P. neurantha*; only two new odorants (dibutyl phthalate and acetic acid, 2-methylpropyl ester) were added, and their odor intensity was less than 1.5, which contributed little to the overall odor formation. UV lacquer-coated *P. neurantha* released the fewest odor compounds. After being coated with UV lacquer, the concentration of most compounds decreased, except for benzene and toluene. The concentration of these two compounds increased 10.38 and 391.68 µg·m^−3^, respectively. As the main odor contributor, toluene increased most significantly, which greatly affected the formation of the overall odor. The odorous compounds of hexanal and benzaldehyde from *P. neurantha* disappeared after lacquering with UV.

**Figure 2:**
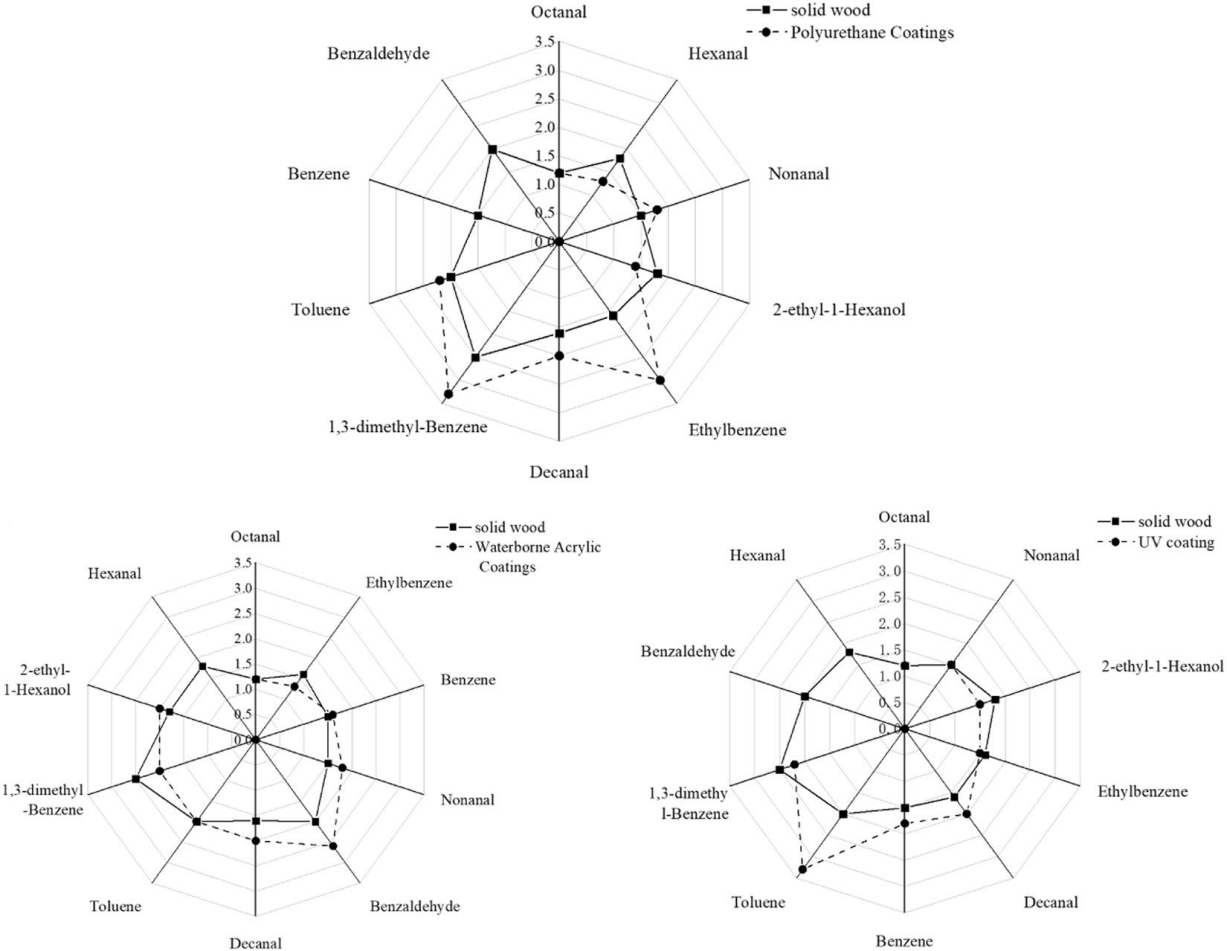
Contrast of odor profile from *P. neurantha (Hemsl.) Gamble* with three lacquer coatings.

To further explore the effects of the three types of lacquer on the odor compounds of *P. neurantha*, the odor intensities of 10 odor compounds released by *P. neurantha* before and after lacquering were compared (Fig. 2), and the effects of various types of lacquer on specific odor compounds were analyzed.

Benzaldehyde, toluene, and 1,3-dimethylbenzene had a high odor intensity among the odor-contributing compounds of unpainted *P. neurantha*, which played a decisive role in the formation of overall odor. Benzene and benzaldehyde had not been detected in the compounds from *P. neurantha* decorated with PU lacquer, indicating PU lacquer had a good sealing effect on these two odors compounds. In addition, the emission of hexanal and 2-ethyl-1-hexanol decreased to less than 1.5 compared with other odor compounds, so these two odor compounds had little effect on the formation of overall odor. In addition to these four odor compounds, the odor intensity of other compounds strengthened. Among them, the odor intensity of ethylbenzene and 1,3-dimethylbenzene was no less than 3, which played a key role in the formation of overall odor. It also released many special odor compounds with a high odor intensity. As a result, the overall characteristic odor and odor intensity of *P. neurantha* decorated with PU lacquer would change significantly.

The odor characteristic compound hexanal was not detected after *P. neurantha* was decorated with waterborne lacquer. In addition, the odor intensity of 1,3-dimethylbenzene and ethylbenzene decreased. Therefore, waterborne lacquer has a good inhibitory effect on the release of these three odor characteristic compounds. However, the VOCs released by waterborne lacquer increased the concentration of benzene, benzaldehyde, 2-ethyl-1-hexanol, decanal, and nonanal; likewise, the odor intensity also increased. Therefore, the overall odor intensity of *P. neurantha* after waterborne lacquer decoration would be enhanced to some extent, but the overall odor characteristics showed little difference.

UV lacquer had a certain inhibitory effect on the odor release of *P. neurantha*. In addition, UV lacquer did not release other types of odor compounds. After being decorated with UV lacquer, hexanal and benzaldehyde were no longer detected. The odor intensity of 1,3-dimethylbenzene, 2-ethyl-1-hexanol, and ethylbenzene also decreased. Decanal and benzene had slightly increased odor intensity, whereas toluene’s odor intensity increased significantly, reaching 3.3, which became the main contributor to the overall odor formation. From the point of view of overall odor formation, the overall odor characteristics of UV lacquer tended to be aromatic, the overall odor intensity changed slightly, and the main odor characteristics and overall odor intensity were caused by the high concentration of toluene.

### Effect of different lacquer decorations on the odor of *P. neurantha*

**Figure 3:**
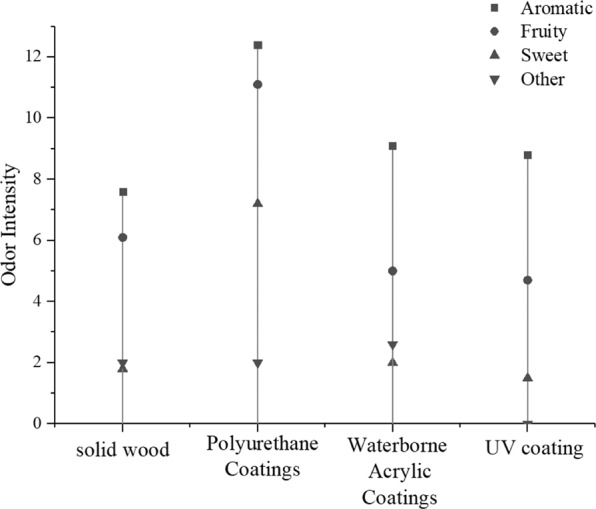
Relation diagram of odor concentration and odor intensity of four boards.

To explore the effect of the three lacquer coatings on the overall odor of *P. neurantha*, the characteristic odors were divided into four categories: aromatic, fruity, sweet, and other. Considering the complex interaction among various odorant compounds, the effect of fusion on the total odor intensity was chosen for this experiment. The total relative intensity of each type was calculated by adding together the intensities of different odorants with similar characteristics. The changes of odor after lacquer decoration are analyzed in Fig. 3. Aromatic was the dominant odor impression of *P. neurantha*, with a rating of 7.6, followed by fruity (6.1). The attributes other (2.0) and sweet (1.8) were rated with low intensities. The total intensity of *P. neurantha* was 17.5.

The overall odor intensity of PU-lacquered *P. neurantha* was the highest among the three lacquered boards, with a total intensity of 32.7. Aromatic (12.4) and fruity (11.1) were the main odors. The intensity of sweet was 7.2, whereas the intensity of other was 2.0. Compared with unpainted *P. neurantha*, the intensity of aromatic and fruity increased by nearly 5.0, which became the decisive odor in overall odor formation, and the intensity of sweet increased by 5.4 and played an important role in modifying the overall odor. The overall odor intensity of the boards was significantly enhanced after PU lacquer decoration, and the overall odor characteristics of PU-lacquered *P. neurantha* were significantly different.

The main characteristic of waterborne-lacquered *P. neurantha* was aromatic, with an intensity of 9.1; the attributes of fruity, other, and sweet were 5.0, 2.6, and 2.0, respectively. Compared with unpainted *P. neurantha*, the overall odor intensity increased slightly. Among them, the intensity of aromatic odor increased by 1.5, becoming the main contributor to the overall odor. The intensity of fruity odor decreased after decoration, but it still played an important role in modifying the formation of the overall odor. Sweet odor and other odors increased, but the intensity of fruity odor was still weak, which had little effect on the overall odor. The waterborne lacquer had little influence on the overall odor characteristics of *P. neurantha*, but the overall odor intensity was enhanced slightly (18.7).

Aromatic was the key characteristic odor of the whole odor of *P. neurantha* varnished by UV lacquer. Compared with unpainted *P. neurantha*, the intensity of the other three types of odor characteristics decreased. Among them, the other category of odor intensity reduced to 0, the total sweet intensity reduced to 1.5, and the fruity intensity still had some modifying effect on the overall odor. Toluene was the main odor contributing compound. UV lacquer would have a good effect on TVOC and odor inhibition when the release of toluene was reduced.

## Conclusion

With the problem of odor caused by solid wood and its lacquer finish is becoming increasingly serious. Studying the VOC and odor characteristic compounds released from lacquer wood can improve the environmental protection level of products and help solve the odor problem of furniture. In this study, GC-MS/O was used to explore the VOC and odor changes of *P. neurantha* after three types of lacquer coatings were applied. Combined with the research results of other scholars, the influence of lacquer decoration on *P. neurantha* was analyzed from many aspects.

1. After being decorated with one of the three lacquers, the release of VOCs from the solid wood could be inhibited by the surface lacquer; however, at the same time, the lacquer material would release some other compounds. The TVOC release of *P. neurantha* increased after one of the three types of lacquer coatings was applied, and the increase of *P. neurantha* with PU lacquer decoration was the most significant.
2. The three lacquers had strong inhibitory effects on the release of olefins from *P. neurantha* and could reduce olefins by more than 90%. PU lacquer could release numerous esters and aromatic hydrocarbons, but it could inhibit aldehydes and ketones. Waterborne lacquer also released numerous esters, resulting in a sharp increase in the release of alcohols after lacquering. UV lacquer had the greatest inhibitory effect on olefins, alcohols, aldehydes, ketones, and other compounds in *P. neurantha* and did not release esters or other compounds, but its toluene release increased sharply.
3. Ten characteristic odor compounds were released from unpainted *P. neurantha*, among which benzaldehyde, toluene, and 1,3-dimethylbenzene played a decisive role in overall odor formation. The overall odor composition of unpainted *P. neurantha* was mainly aromatic. After finishing with PU lacquer, the overall odor intensity of *P. neurantha* was significantly enhanced, and the overall odor was mainly fruity and aromatic. The types of odor compounds released by waterborne lacquer-coated *P. neurantha* were similar to those of unpainted *P. neurantha*, but the overall odor intensity was slightly enhanced. The overall odor of *P. neurantha* with UV lacquer was basically aromatic.

## Methods

### Materials

*P. neurantha* (Hemsl.) Gamble, produced on the GuangYun Forest Farm of Guilin City, Guangxi, China was selected as the test material. The diameter of the disc was 60 mm, and the moisture content was 12%. The test base material was coated uniformly with coatings of PU lacquer, waterborne lacquer, and UV-curable lacquer. Specific finishing parameters were as follows. PU coatings (Huarun transparent primer/semiclear finish, main agent–curing agent–diluent ratio = 2:1:1): two primers (10 m^2^/kg) and two topcoats (10 m^2^/kg) were each lacquered 12 h apart. Waterborne acrylic paint (Sankeshu 360 waterborne wood paint–transparent primer/varnish, main agent–water ratio = 10:1): two primers (10 m^2^/kg) and two topcoats (10 m^2^/kg/time) were each lacquered 12 h apart. UV-curable coatings (plain chemical–light-emitting diode UV curing varnish, spray-gun cleaning product, surface-spraying UV coating 55 and leveling for 3–10 min of UV curing): a total of two coatings (10 m^2^/kg/time) were applied. Construction environment conditions: indoor temperature was 23 °C ± 2 °C, and relative humidity was 40% ± 10%. The room was in a continuous ventilation state. The surface of the solid wood was polished with 150-mesh sandpaper, and 180-mesh sandpaper was used between the two paint applications. After finishing the coating, the sample was cut into a circle with a diameter of 60 mm. The edge of the sample was sealed with aluminum tape along the thickest portion to prevent high release from the edge of the material. After edge sealing, the material was vacuum sealed, a paper label was affixed, and the sample was stored in a refrigerator at −30 °C.

### Equipment

#### Sampling equipment

**Figure 4:**
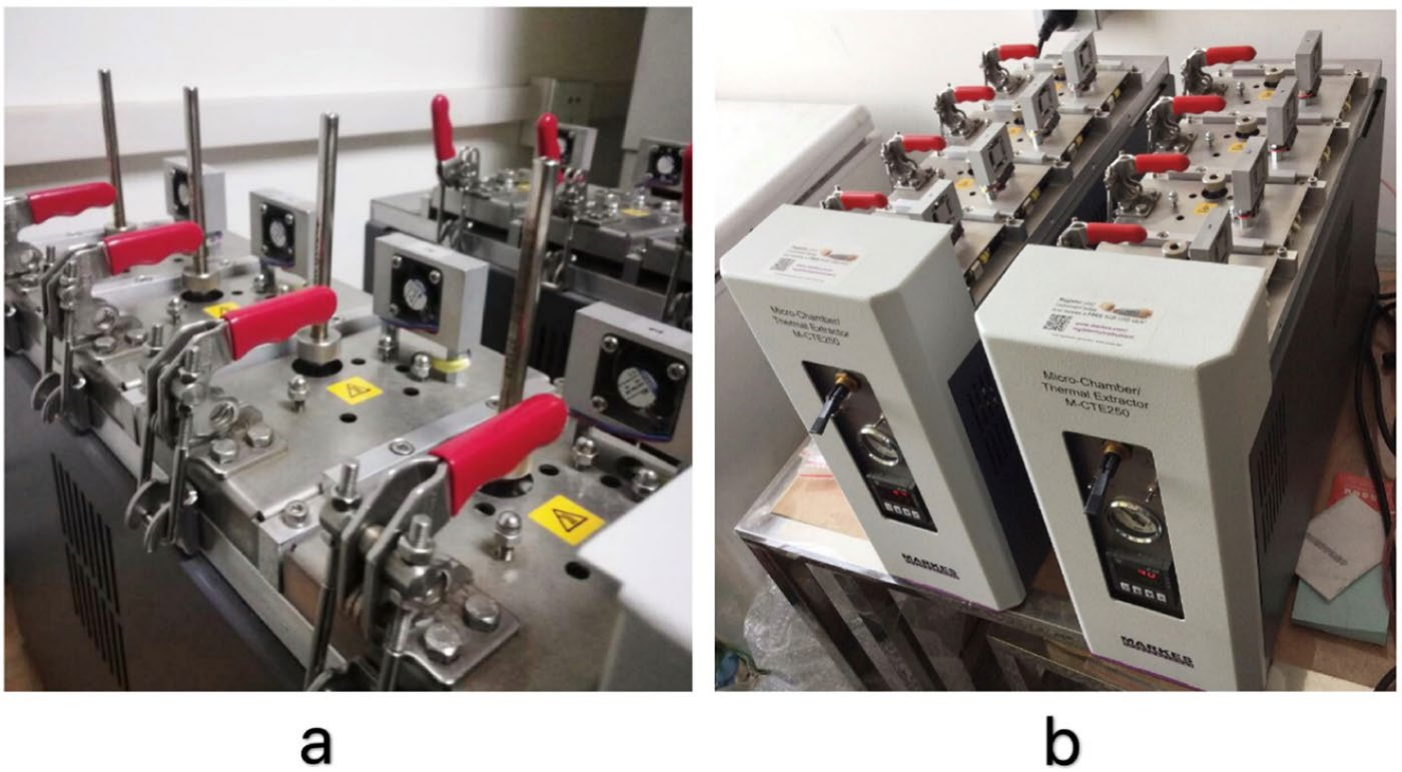
Photograph of sampling equipment. (**a)**: Tenax-TA adsorption tubes in sampling; (**b**): Microchamber thermal extractor.

The microchamber thermal extractor and Tenax-TA adsorption tubes (Fig. 4) was produced by Markes International of the United Kingdom (model M-CTE250). Nitrogen was used as the carrier gas, and the temperature could be adjusted in the range of 0 °C–250 °C. The tube length was 89 mm, the outer diameter was 6.4 mm, and it contained 2,6-diphenylfuran porous polymer resin filler. The two ends were equipped with copper caps, which could effectively adsorb VOCs volatilized from wood-based panels and store them in the tubes.

#### Analysis and detection equipment

The thermal desorption instrument was produced by Markes (Unity model). The cold trap adsorption temperature was −15 °C, the carrier gas was helium, the carrier gas flow rate was 30 mL/min, the analytical temperature was 300 °C, the pipeline temperature was 180 °C, the thermal desorption sample took 10 min, and the prepurging time was 1 min.

The DSQ single four-stage gas chromatography–mass spectrometry (GC-MS) instrument was produced by Thermo Company of the United States. The instrument chromatographic column type was DB-5, with the following specifications: 3000 mm × 0.26 mm × 0.25 µm quartz capillary column, GC inlet temperature of 250 °C, carrier gas flow rate of 1.0 mL/min (constant current), and nonshunt injection. The heating procedure was as follows: use a start temperature of 40 °C, keep increasing 2 °C/min, rise to 50 °C, keep steady at 4 min, and then rise to 150 °C/min. The temperature was then held for 4 min, and finally increased 10 °C/min to 250 °C for 8 min. The ionization mode was the ionization source (EI) with 70 eV of energy, a 230 °C ion source temperature, a 250 °C transmission line temperature, a 50- to 450-amu scanning mode, a 280 °C interface temperature, and a 150 °C four-stage rod temperature.

The Olfactory Detector Sniffer 9100 came from Brechbühler (Echallens, Switzerland). The transmission line temperature was 150 °C, and nitrogen was used as the carrier gas through a purge valve. Moist air was added to prevent dehydration of the nasal mucosa of the odor assessors. Direct intensity methods were chosen for analysis of the compounds.

The TP-5000 universal thermal desorptioner was produced by Beifen Tianpu Instrument Technology Co. in Beijing. It can desorb and remove the residues from the Tenax tube after sample analysis.

## Methods

### Sampling

Before the experiment, the microchamber thermal extractor was cleaned once with deionized water and once with methanol. The samples were put in the microchamber thermal extractor under the specific sampling condition. The area of exposure was 5.65 × 10^−3^ m^3^, the cell volume was 1.35 × 10^−4^ m^3^, and the loading rate was 41.85 m^2^·m^−3^. Four specimens were made for an identity condition during a sampling cycle of 8 h. The environment condition was as follows: temperature of 23 °C ± 5 °C, relative humidity of 30% ± 10%, ratio of the air exchange rate to the loading factor of 0.5 m^3^·m^−2^·h^−1^. Then, 2 L of VOCs were collected by the microchamber thermal extractor. The adsorption tubes that collected the samples were covered with copper caps, wrapped in polytetrafluoroethylene bags, labeled, and stored in a freezer at −30 °C. Four samples were made for each type of board (three repetitions were performed for each measurement).

### GC-MS analysis method

The external standard method was used in this experiment. The compounds were quantified according to the Chinese national standard GB/T 29899-2013^37^, and the data processing was completed using the Xcalibur software system. Qualitative volatile compounds were identified by the U.S. National Institute of Standards and Technology (NIST 08 standard library) and the Wiley library, and only the positive and negative matching degrees of more than 750 were used (the maximum value was 1000). Through an Excel-based data processing system, the relative percentage content of each chemical component in wood odor substances was obtained by the area normalization method.

### GC-O analysis method

**Figure 5:**
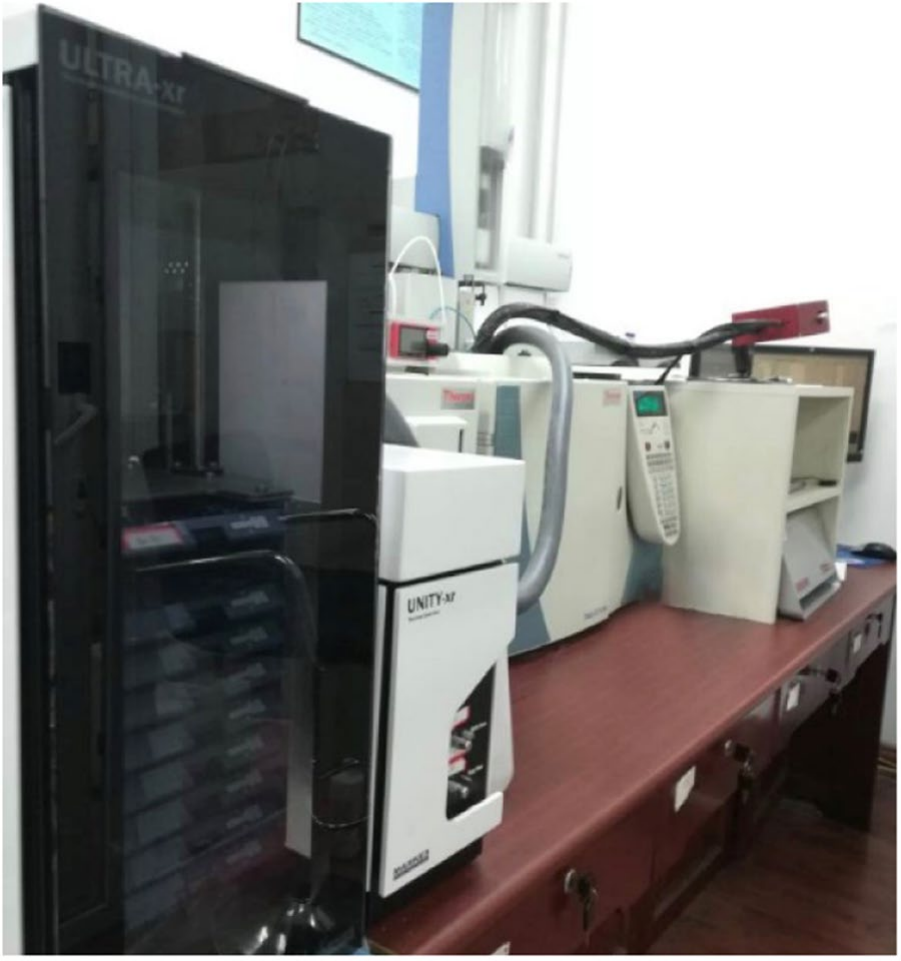
GC/MS-O operating environment.

GC-O analysis used the time-intensity method^38^. After the peak of the detected substance indicated the composition and concentration of the compound, the odor characteristics and intensity of the odor emitted from the chromatographic column were recorded by the evaluator’s sniffing. Five grades of odor intensity were set, which ranged from 0 to 4: 0 to denote no odor, 1 to denote weak odor intensity, 2 to denote moderate odor intensity, 3 to denote strong odor intensity, and 4 to denote the strongest odor intensity^39^. Based on specific screening and training recommendations in the standard ISO 12219-7:2017^40^, four panelists (who were between 20 and 30 years old, had good olfactory perception, were nonsmokers, did not use heavily fragrant cosmetics, had a nonallergic constitution, and did not suffer chronic rhinitis) were selected to carry out the experiment. After screening and training, they were familiarized with various odor compounds in wood, including understanding the odor characteristics wood, learning the intensity evaluation methods, and accumulating commonly used odor description vocabulary. Before the experiment, all panelists were trained in smelling fragrance. Following the National Standards Authority of Ireland standard EN 13725-2003^41^, the olfactory discrimination test was conducted in a room with good ventilation conditions, a temperature of 20 °C–25 °C, and a relative humidity of 40%. It was required that the room have no peculiar smell. The operating environment of GC/MS-O was showed in Fig. 5. Each sample was sniffed twice by each panelist. When the test results were collated and recorded, the same odor characteristic descriptions obtained by at least two panelists for the same sample were recorded in the results. The average odor intensity results of the four panelists were taken as the intensity values. The compounds were identified by aroma odor recognition and odor description.

### Ethical statement

The study was conducted in agreement with the Declaration of Helsinki. The research contents and methods as mentioned above were evaluated and approved by College of Materials Science and Engineering (Ethics committee), Northeast Forestry University. Informed consent was obtained from all subjects participating in the study.

## Supplementary information

Appendix 1.

Appendix 2.

Appendix 3.

Appendix 4.

Supplementary information.
