# Supplementary material for: Effect of lacquer decoration on VOCs and odor release from P. neurantha (Hemsl.) Gamble
Source: Sci Rep. 2020 Jun 12;10:9565. doi: 10.1038/s41598-020-66724-0 (PMC7293346; doi:10.1038/s41598-020-66724-0)
Supplement: Supplementary file 1 — Appendix 1. [file 41598_2020_66724_MOESM1_ESM.docx]

| Appendix 1. Mass concentration of VOCs compounds released from solid wood | | | | | | | |
| --- | --- | --- | --- | --- | --- | --- | --- |
|  | Molecular Formula | Compound Name | Mass Concentration /ug·m^-3^ |  | Molecular Formula | Compound Name | Mass Concentration /ug·m^-3^ |
| Alenes | C_6_H_6_ | Benzene | 12.8192 | Olefins | C_15_H_24_ | à-Cubebene | 134.9113 |
|  | C_7_H_8_ | Toluene | 11.6758 |  | C_15_H_24_ | Caryophyllene | 104.5338 |
|  | C_8_H_10_ | Ethylbenzene | 25.3815 |  | C_15_H_24_ | à-Caryophyllene | 14.6494 |
|  | C_8_H_10_ | 1,3-dimethyl-Benzene | 93.3388 | Aldehyde | C_6_H_12_O | Hexanal | 9.2318 |
|  | C_15_H_24_ | octahydro-7-methyl-3-methylene-4-(1-methylethyl)-, [3aS-(3aà,3bá,4á,7à,7aS*)]-1H-Cyclopenta[1,3]cyclopropa[1,2]benzene | 9.2654 |  | C_7_H_6_O | Benzaldehyde | 4.9284 |
|  | C_15_H_24_ | 1,2,3,5,6,8a-hexahydro-4,7-dimethyl-1-(1-methylethyl)-, (1S-cis)-Naphthalene | 42.2135 |  | C_8_H_16_O | Octanal | 4.5176 |
| Alkane | C_12_H_26_ | 3-methyl-Undecane | 5.2939 |  | C_9_H_18_O | Nonanal | 6.2410 |
|  | C_15_H_32_ | Pentadecane | 4.9963 |  | C_10_H_20_O | Decanal | 5.8315 |
|  | C_16_H_34_ | Hexadecane | 6.4295 | other | C_16_H_32_O_2_ | n-Hexadecanoic acid | 49.5443 |
| alcohol | C_8_H_18_O | 2-ethyl-1-Hexanol | 11.5193 |  |  |  |  |
