# Supplementary material for: Effect of lacquer decoration on VOCs and odor release from P. neurantha (Hemsl.) Gamble
Source: Sci Rep. 2020 Jun 12;10:9565. doi: 10.1038/s41598-020-66724-0 (PMC7293346; doi:10.1038/s41598-020-66724-0)
Supplement: Supplementary file 2 — Appendix 2. [file 41598_2020_66724_MOESM2_ESM.docx]

| Appendix 2. Mass concentration of VOCs compounds released from Polyurethane lacquer | | | | | | | |
| --- | --- | --- | --- | --- | --- | --- | --- |
|  | Molecular Formula | Compound Name | Mass Concentration /ug·m^-3^ |  | Molecular Formula | Compound Name | Mass Concentration /ug·m^-3^ |
| Arenes | C_14_H_10_ | Phenanthrene | 10.1120 | alcohol | C_6_H_12_O | 2-ethyl-Cyclobutanol | 5.7164 |
|  | C_9_H_10_ | Indane | 8.1316 |  | C_8_H_18_O | 2-ethyl-1-Hexanol | 5.9694 |
|  | C_7_H_8_ | Toluene | 16.3181 |  |  |  |  |
|  | C_8_H_10_ | Ethylbenzene | 176.5671 | Aldehyde | C_6_H_12_O | Hexanal | 6.6422 |
|  | C_8_H_10_ | 1,3-dimethyl-Benzene | 633.8477 |  | C_8_H_16_O | Octanal | 5.2478 |
|  | C_8_H_10_ | o-Xylene | 240.1235 |  | C_9_H_18_O | Nonanal | 9.0897 |
|  | C_9_H_12_ | 1-ethyl-4-methyl-Benzene | 7.8884 |  | C_10_H_20_O | Decanal | 7.3752 |
|  | C_9_H_12_ | propyl-Benzene | 13.2138 |  |  |  |  |
|  | C_9_H_12_ | 1-ethyl-3-methyl-Benzene | 37.8599 | Ester | C_16_H_22_O_4_ | Dibutyl phthalate | 7.4099 |
|  | C_9_H_12_ | 1,3,5-trimethyl-Benzene | 16.2397 |  | C_16_H_22_O_4_ | 1,2-Benzenedicarboxylic acid, mono(2-ethylhexyl) ester | 8.5743 |
|  | C_9_H_12_ | 1-ethyl-2-methyl-Benzene | 23.3442 |  | C_7_H_14_O_2_ | 2-Butanol, 3-methyl-, acetate | 7.7901 |
|  | C_9_H_12_ | 1,2,3-trimethyl-Benzene | 73.0694 |  | C_6_H_12_O_2_ | Acetic acid, 1-methylpropyl ester | 43.8613 |
|  | C_10_H_14_ | 2-ethyl-1,4-dimethyl-Benzene | 5.0852 |  | C_6_H_12_O_2_ | Acetic acid, 2-methylpropyl ester | 8.3519 |
|  | C_10_H_8_ | 1-methylene-1H-Indene, | 4.9429 |  | C_6_H_12_O_2_ | Acetic acid, butyl ester | 273.7115 |
|  | C_16_H_18_ | 2,2',5,5'-tetramethyl-1,1'-Biphenyl | 6.0536 |  | C_7_H_14_O_2_ | 3-methyl-,2-Butanol, acetate | 6.8742 |
| Alkane | C_8_H_16_ | ethyl-Cyclohexane | 4.9992 |  | C_7_H_14_O_2_ | 2-Pentanol, acetate | 17.7815 |
|  | C_12_H_26_ | 2,2-dimethyl-Decane | 7.5155 |  | C_6_H_12_O_3_ | 1-Methoxy-2-propyl acetate | 32.3178 |
|  | C_14_H_30_ | Tetradecane | 4.7366 |  | C_7_H_12_O_4_ | Pentanedioic acid, dimethyl ester | 17.5278 |
|  | C_16_H_34_ | Hexadecane | 6.3457 |  | C_8_H_14_O_4_ | Hexanedioic acid, dimethyl ester | 11.9296 |
| Olefins | C_10_H_16_ | 3-Carene | 5.166367 | other | C_15_H_24_O | Butylated Hydroxytoluene | 10.82322 |
|  | C_7_H_8_ | 2-propenylidene-Cyclobutene | 11.90589 |  | C_14_H_22_O | (octyloxy)-Benzene | 4.851479 |
|  | C_9_H_10_ | Deltacyclene | 6.843332 |  | C_18_H_26_O_3_ | 2-Ethylhexyl trans-4-methoxycinnamate | 15.28035 |
