# Supplementary material for: Effect of lacquer decoration on VOCs and odor release from P. neurantha (Hemsl.) Gamble
Source: Sci Rep. 2020 Jun 12;10:9565. doi: 10.1038/s41598-020-66724-0 (PMC7293346; doi:10.1038/s41598-020-66724-0)
Supplement: Supplementary file 3 — Appendix 3. [file 41598_2020_66724_MOESM3_ESM.docx]

| Appendix 3. Mass concentration of VOCs compounds released from waterborne acrylic lacquer | | | | | | | |
| --- | --- | --- | --- | --- | --- | --- | --- |
|  | Molecular Formula | Compound Name | Mass Concentration /ug·m^-3^ |  | Molecular Formula | Compound Name | Mass Concentration /ug·m^-3^ |
| Arenes | C_6_H_6_ | Benzene | 18.2939 | Aldehyde | C_10_H_20_O | Decanal | 8.1977 |
|  | C_7_H_8_ | Toluene | 12.3449 |  | C_7_H_6_O | Benzaldehyde | 5.1650 |
|  | C_8_H_10_ | Ethylbenzene | 16.5416 |  | C_8_H_16_O | Octanal | 5.6745 |
|  | C_8_H_10_ | 1,3-dimethyl-Benzene | 49.6477 |  | C_9_H_18_O | Nonanal | 8.6742 |
|  | C_10_H_8_ | 1-methylene-1H-Indene | 4.9887 | Ester | C_12_H_24_O_3_ | Propanoic acid, 2-methyl-, 2,2-dimethyl-1-(2-hydroxy-1-methylethyl)propyl ester | 6.8260 |
| Alkane | C_8_H_16_ | ethyl-Cyclohexane | 4.2973 |  | C_16_H_22_O_4_ | Dibutyl phthalate | 4.7528 |
|  | C_14_H_30_ | Tetradecane | 5.2744 |  | C_12_H_24_O_3_ | Propanoic acid, 2-methyl-, 3-hydroxy-2,4,4-trimethylpentyl ester | 9.6830 |
| Olefins | C_10_H_16_ | Bicyclo[3.1.1]hept-2-ene, 3,6,6-trimethyl- | 5.7175 |  | C_10_H_10_O_4_ | Dimethyl phthalate | 5.5886 |
|  | C_15_H_24_ | Copaene | 5.3954 |  | C_16_H_30_O_4_ | Propanoic acid, 2-methyl-, 1-(1,1-dimethylethyl)-2-methyl-1,3-propanediyl ester | 494.5123 |
| alcohol | C_6_H_14_O_3_ | 3,3'-oxybis-1-Propanol | 87.0201 |  | C_16_H_30_O_4_ | Pentanoic acid, 2,2,4-trimethyl-3-carboxyisopropyl, isobutyl ester | 4.8936 |
|  | C_8_H_18_O | 2-ethyl-1-Hexanol | 8.7467 | other | C_8_H_16_O_3_ | 2,4-Diethyl-6-methyl-1,3,5-trioxane | 6.8827 |
|  | C_6_H_14_O_3_ | 2,2'-oxybis-1-Propanol | 59.9400 |  | C_12_H_24_O_2_ | Dodecanoic acid | 6.8590 |
