# Supplementary material for: Effect of lacquer decoration on VOCs and odor release from P. neurantha (Hemsl.) Gamble
Source: Sci Rep. 2020 Jun 12;10:9565. doi: 10.1038/s41598-020-66724-0 (PMC7293346; doi:10.1038/s41598-020-66724-0)
Supplement: Supplementary file 4 — Appendix 4. [file 41598_2020_66724_MOESM4_ESM.docx]

| Appendix 4. Mass concentration of VOCs compounds released from UV lacquer | | | | | | | |
| --- | --- | --- | --- | --- | --- | --- | --- |
|  | Molecular Formula | Compound Name | Mass Concentration /ug·m^-3^ |  | Molecular Formula | Compound Name | Mass Concentration /ug·m^-3^ |
| Arenes | C_6_H_6_ | Benzene | 23.1977 | Olefins | C_8_H_16_ | 3-methyl-2-Heptene, | 5.5755 |
|  | C_7_H_8_ | Toluene | 403.3592 |  | C_10_H_16_ | 2,6,6-trimethyl-, (ñ)-Bicyclo[3.1.1]hept-2-ene | 5.7702 |
|  | C_8_H_10_ | Ethylbenzene | 26.5617 | alcohol | C_8_H_18_O | 2-ethyl-1-Hexanol | 6.9941 |
|  | C_8_H_10_ | 1,3-dimethyl-Benzene | 74.6074 | Aldehyde | C_9_H_18_O | Nonanal | 5.9350 |
|  | C_9_H_12_ | 1,3,5-trimethyl-Benzene | 4.8334 |  | C_10_H_20_O | Decanal | 5.4382 |
|  | C_10_H_8_ | 1-methylene-1H-Indene | 4.6961 |  | C_7_H_6_O | Benzaldehyde | 5.7843 |
| Alkane | C_8_H_16_ | ethyl-Cyclohexane | 6.5439 |  | C_8_H_16_O | Octanal | 5.4471 |
|  | C_12_H_26_ | 2,2,7,7-Tetramethyloctane | 6.4614 | other | C_13_H_16_O_2_ | (1-hydroxycyclohexyl) phenyl-Methanone | 11.6350 |
|  | C_14_H_30_ | Tetradecane | 4.7602 |  |  |  |  |
|  | C_16_H_34_ | Hexadecane | 5.1574 |  |  |  |  |
