# Supplementary material for: Effect of lacquer decoration on VOCs and odor release from P. neurantha (Hemsl.) Gamble
Source: Sci Rep. 2020 Jun 12;10:9565. doi: 10.1038/s41598-020-66724-0 (PMC7293346; doi:10.1038/s41598-020-66724-0)
Supplement: Supplementary file 5 — Supplementary information. [file 41598_2020_66724_MOESM5_ESM.doc]

1. Title: Effect of lacquer decoration on VOCs and odor release from *P. neurantha* (Hemsl.) Gamble
2. Authors names and affiliations:

Qifan Wang,^1,+^ Bin Zeng,^1,+^ Jun Shen,^1,*^ & Huiyu Wang^1^

^+^Qifan Wang and Bin Zeng contributed equally to this work.

Authors affiliations:

^1^. College of Material Science and Engineering, Northeast Forestry University, Harbin, China

First author: Qifan Wang and Bin Zeng

*Corresponding author: Shen Jun, Ph.D., College of Material Science and Engineering, Northeast Forestry University, Harbin, China. Main research direction: wood science and technology.

*Corresponding email: shenjunr@126.com;

Mailing address: Northeast Forestry University, 26 Hexing Road, Harbin 150040, China
